# Dengue and Zika virus NS4B proteins differ in topology and in determinants of ER membrane protein complex dependency

**DOI:** 10.1128/jvi.01443-24

**Published:** 2024-12-31

**Authors:** Samuel S. Porter, Talon M. Gilchrist, Samantha Schrodel, Andrew W. Tai

**Affiliations:** ^1^Division of Gastroenterology, Department of Internal Medicine, University of Michigan Medical School 12266 , Ann Arbor, Michigan, USA; 2 Department of Microbiology and Immunology, University of Michigan Medical School 12266 , Ann Arbor, Michigan, USA; 3 Medicine Service, VA Ann Arbor Healthcare System 20034 , Ann Arbor, Michigan, USA; Wake Forest University School of Medicine, Winston-Salem, North Carolina, USA

**Keywords:** virology, flavivirus, Dengue fever, Zika

## Abstract

Flaviviruses utilize the cellular endoplasmic reticulum (ER) for all aspects of their lifecycle. Genome replication and other viral activities take place in structures called replication organelles (ROs), which are invaginations induced in the ER membrane. Among the required elements for RO formation is the biogenesis of viral nonstructural proteins NS4A and NS4B. We have previously shown that NS4A and NS4B from Dengue virus (DENV) and Zika virus (ZIKV) depend on the cellular ER membrane protein complex (EMC) for biogenesis. Here, we find that this dependency extends to the NS4A and NS4B proteins of Yellow Fever virus (YFV) and West Nile virus (WNV), which share similar computationally predicted membrane topologies. However, we demonstrate that ZIKV NS4B has different determinants of its dependency on the EMC than those for DENV NS4B, as well as a different membrane topology. Furthermore, we characterize mutant isolates of DENV and ZIKV that were serially passaged in EMC knockout cells and find that none are completely independent of the EMC for infection, and that mutant NS4B proteins remain sensitive to EMC depletion, suggesting a high genetic barrier to EMC depletion. Collectively, our findings are consistent with a model in which the EMC recognizes multiple determinants in the NS4B protein to support infection in several flaviviruses of critical public health importance.

**IMPORTANCE:**

The NS4A and NS4B proteins of flaviviruses are critically important to replication, but little is known about their function. It has been previously reported that the cellular EMC supports the biogenesis of NS4A and NS4B from Dengue and Zika virus. In this work, we demonstrate that this dependency on the EMC for NS4A and NS4B biogenesis extends to the West Nile and Yellow Fever viruses. Furthermore, we examine the features of ZIKV NS4B and find that its membrane topology of ZIKV NS4B and its determinants of dependency on the EMC are different from those previously described in DENV NS4B. Finally, we present evidence that there is a high genetic barrier for Dengue and Zika viruses to overcome EMC depletion.

## INTRODUCTION

Viruses of the family *Flaviviridae*, including Dengue (DENV), Zika (ZIKV), Yellow Fever (YFV), and West Nile (WNV) viruses, are among the most severe infectious burdens to human health globally. These mosquito-transmitted viruses infect hundreds of millions of individuals each year resulting in tens of thousands of deaths annually. Due to rising surface temperatures, the latitudes at which the mosquito vectors for Dengue and Zika viruses (*Aedes aegypti* and *albopictus,* respectively) can breed are continually inching northward, leading to the increased threat of local transmission in the continental United States in the coming years. Neither of these viruses currently have antiviral therapies nor a FDA-approved vaccine for naïve individuals.

After entry into the host cell, the positive strand RNA genome of flaviviruses is translated at the endoplasmic reticulum (ER) into a single polyprotein that is co-translationally cleaved into 10 individual viral proteins. Of these, seven are nonstructural proteins, including NS4A and NS4B. While their exact mechanistic functions are not fully understood, NS4A and NS4B are essential for viral genome replication and are thought to be involved in the formation of viral replication organelles (ROs) by inducing invagination in the ER membrane (1, 2).

Our laboratory and others have identified the ER membrane complex (EMC) as an essential host factor for Dengue and Zika virus infection through its support of NS4A and NS4B protein biogenesis (3, 4), although a precise mechanism for this activity has yet to be elucidated. This 10-subunit cellular protein complex has been shown to function as a chaperone for subsets of ER resident, multipass transmembrane proteins, especially those with weakly or moderately hydrophobic transmembrane domains (5–9). In DENV NS4B, there are five predicted potential transmembrane domains (pTMDs), the first two of which are marginally hydrophobic and have been experimentally demonstrated to not actually cross the lipid bilayer (10). We have previously shown that deletion of these two pTMDs ablates dependency on the EMC for NS4B biogenesis, but it is not known whether this model extends to other flaviviruses (3). Alternatively, a recent report described a DENV isolate passaged in EMC-depleted cell lines that can replicate at WT levels and support NS4B synthesis (4). The mutation in NS4B was not in the domains we had previously demonstrated to confer EMC dependency, so this suggested an alternate model of the determinants of NS4B dependency. Furthermore, it is unclear whether either model would apply to ZIKV NS4B.

Here, we demonstrate that the dependency on the EMC for biogenesis of NS4A and NS4B extends to multiple additional species of flavivirus. We determine the membrane topology of ZIKV NS4B and find that it differs from that of DENV NS4B. Furthermore, we show that ZIKV NS4B has different determinants of EMC dependency than previously established for DENV NS4B. Finally, we evaluate putatively EMC-independent isolates of ZIKV and DENV and find that neither can escape EMC dependence for replication or NS4A and NS4B synthesis.

## RESULTS

### NS4A and NS4B dependence on cellular EMC is conserved in multiple flaviviruses

**Fig 1:**
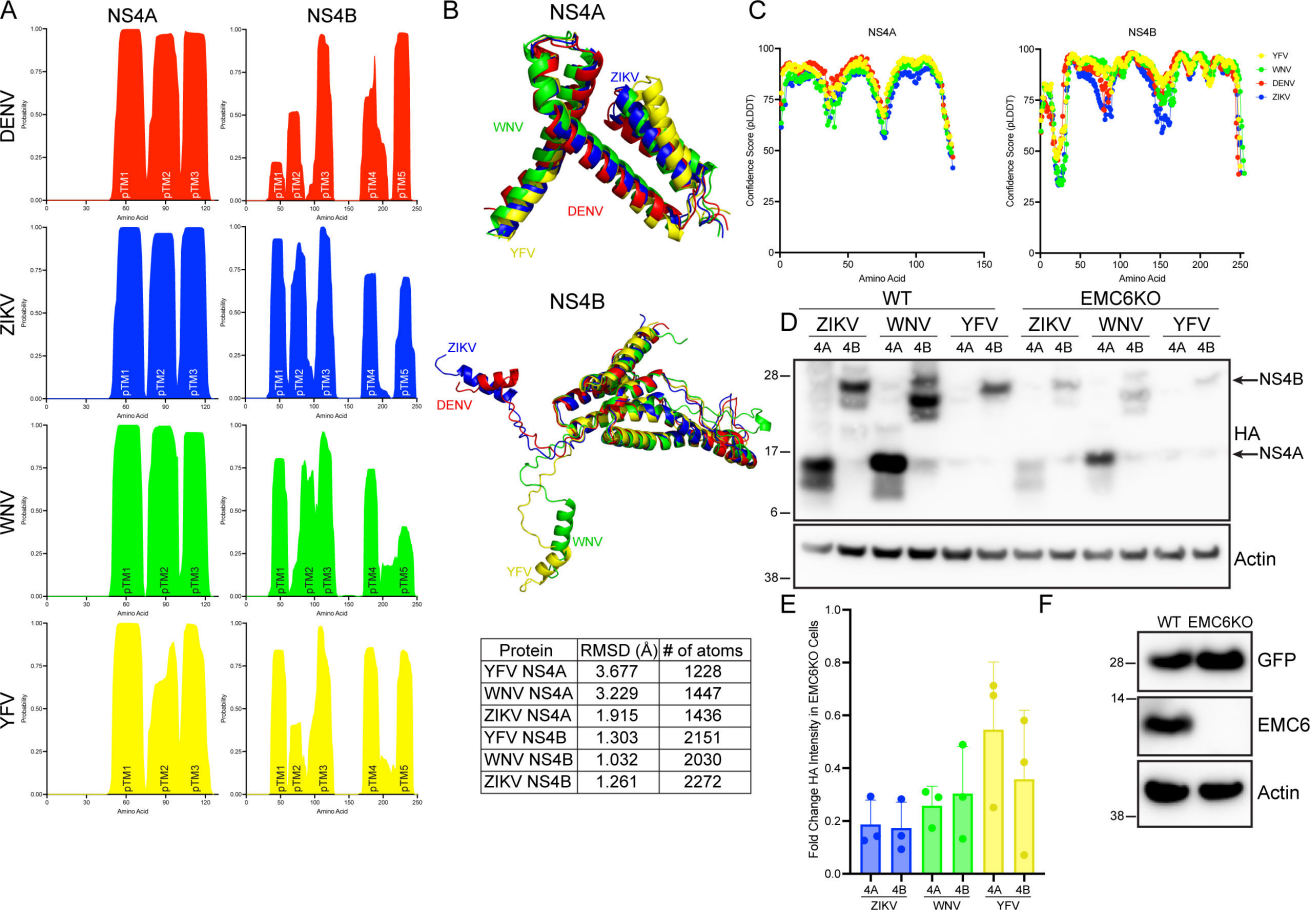
Predicted topologies, structure, and EMC dependence of flavivirus NS4A and NS4B proteins. (**A**) Probability of indicated amino acid being within a transmembrane domain for the indicated flavivirus NS4A and NS4B proteins, based on TMHMM 2.0 hydrophobicity predictions. (**B**) Alignments of AlphaFold2-predicted protein structures of flavivirus NS4A (top) and NS4B (bottom). Table indicates root-mean-square deviation of indicated protein from DENV NS4A- or NS4B-predicted structures. (**C**) Confidence scores for AlphaFold2 predictions. (**D**) Wild-type 293T cells or pooled EMC6 knockout cells were transiently transfected with plasmids encoding NS4A-HA or 2K-NS4B-HA from indicated flavivirus species. Proteins were resolved using SDS-PAGE followed by immunoblotting for the indicated proteins. Blots are representative of three independent experiments. (**E**) Quantitation of D. Bars represent means ± SD. (**F**) Wild-type 293T cells or pooled EMC6 knockout cells were transiently transfected with a plasmid encoding GFP. Proteins were resolved using SDS-PAGE followed by immunoblotting for the indicated proteins. Blots are representative of three independent experiments.

The EMC has been proposed to function as a chaperone for proteins with complex or weakly hydrophobic transmembrane domains (6, 7). We had previously demonstrated that, in DENV NS4B, the partially hydrophobic domains, pTMD1 and pTMD2, confer dependency on EMC (3). TMHMM 2.0 transmembrane domain predictions of NS4A from DENV, ZIKV, YFV, and WNV show that the predicted transmembrane domains (pTMDs) in these proteins are conserved in both location and degree of hydrophobicity (Fig. 1A) (11). For NS4B, each species has the same number of pTMDs but has considerable variation in their degree of hydrophobicity (Fig. 1A). Unlike DENV NS4B, pTMDs 1 and 2 of ZIKV NS4B are strongly hydrophobic, while pTMDs 4 and 5 have lower levels of predicted hydrophobicity. Similarly, NS4B proteins from WNV and YFV have marginally hydrophobic pTMDs at positions 5 and 2, respectively. However, the AlphaFold2-predicted structures of these proteins showed near complete structural conservation of NS4A, while the NS4B structures aligned nearly completely after the first 30 residues (Fig. 1B) (12), with the important caveat that AlphaFold2 was not specifically developed for prediction of transmembrane protein structure. As the N-terminal region of NS4B is highly hydrophilic (Fig. 1A), structural prediction is more difficult, and this is reflected in the reduced confidence scores of that region (Fig. 1C), likely explaining the lack of alignment.

To assess whether the computationally predicted structural similarity of these proteins is associated with conservation of EMC dependence, we generated constructs expressing HA-tagged NS4A and 2K-NS4B from WNV and YFV. We transfected these plasmids (with ZIKV NS4A and NS4B as positive controls for EMC dependency) into wild-type and EMC knockout cells and assessed the steady-state expression level of the viral proteins by immunoblot. The levels of NS4A and NS4B from each of these flaviviruses were significantly reduced in EMC-depleted cells (Fig. 1D and E), while there was no reduction in the expression level of GFP, an EMC-independent protein (Fig. 1F). These data indicate that the dependency on EMC for NS4A and NS4B biogenesis extends to YFV and WNV and may suggest a shared mechanism for EMC dependency.

### Role of partially hydrophobic pTMDs of NS4B in EMC dependency

**Fig 2:**
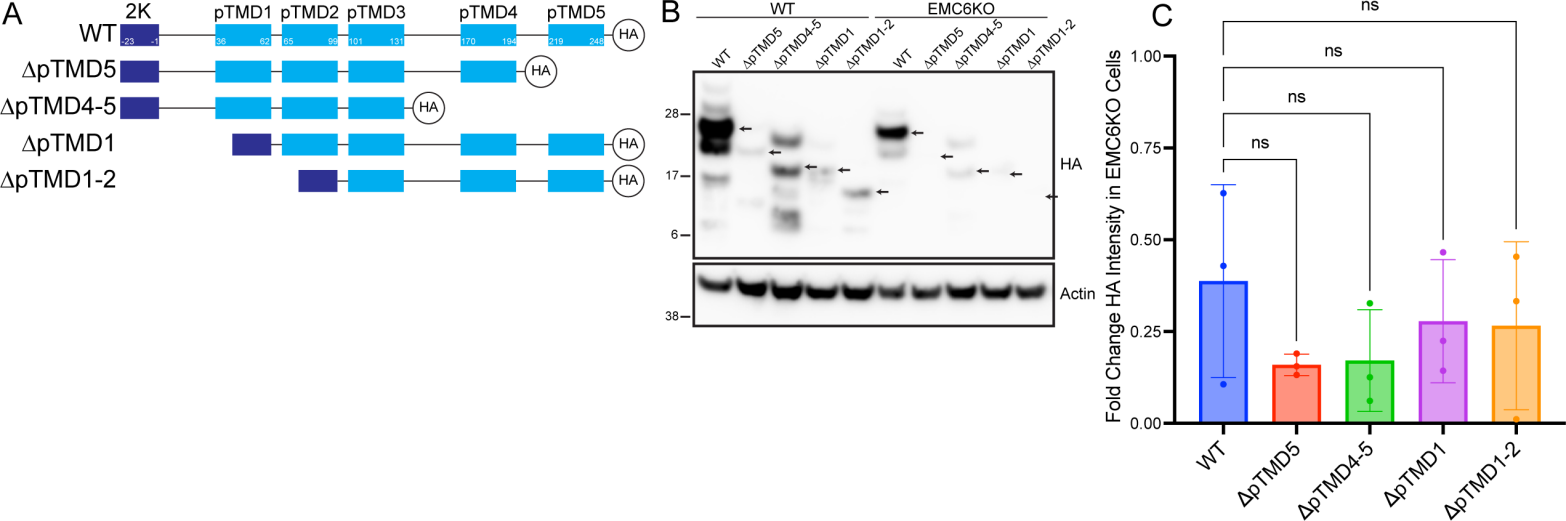
ZIKV NS4B has multiple determinants of EMC dependency. (**A**) Schematic of truncation mutations of ZIKV NS4B. (**B**) Wild-type 293T cells or EMC6 KO cells were transfected to express NS4B-HA truncation mutants. Proteins were resolved using SDS-PAGE followed by Western blotting for the indicated proteins. Arrows indicated NS4B bands of interest. Blots are representative of three independent experiments. (**C**) Quantitation of B. Bars represent means ± SD.

We have previously reported that deletion of the partially hydrophobic pTMD1 and pTMD2 from DENV NS4B ablates dependency on the EMC (3). Given that NS4Bs from multiple species of *Flaviviridae* were dependent on the EMC, we hypothesized that they may share the same mechanism as previously suggested for DENV NS4B. To test this, we created HA-tagged truncation mutants of ZIKV 2K-NS4B with deletions in the pTMD1 + pTMD2 regions (mimicking the regions in our previous DENV NS4B experiments) and the pTMD4 + pTMD5 regions (the TMDs in ZIKV NS4B computationally predicted to be marginally hydrophobic) (Fig. 2A). Interestingly, when these constructs were transfected into cells with and without EMC6 knockout, none demonstrated ablation of EMC dependency (Fig. 2B and C). These results contrast with our previous work with DENV NS4B and suggest that ZIKV NS4B does not share the same determinant(s) of EMC dependency with DENV NS4B and in fact may encode multiple determinants.

### Membrane topology of ZIKV NS4B

We next sought to determine the membrane topology of ZIKV NS4B. Of all the species of flavivirus NS4B, only DENV NS4B has had had its topology experimentally determined (10, 13, 14). In DENV NS4B, the partially hydrophobic pTMDs 1 and 2 do not fully cross the membrane, while the more hydrophobic pTMDs 3–5 do (10). While the computationally predicted 3D structures of ZIKV and DENV NS4B appear to be conserved (Fig. 1B), the computationally predicted membrane topology suggests that ZIKV NS4B pTMDs 1–3 have high probabilities of fully crossing the membrane while pTMDs 4–5 may not (Fig. 1A). We asked if the membrane topology of ZIKV NS4B would be consistent with that of DENV NS4B, the computational model, or a third alternative structure.

**Fig 3:**
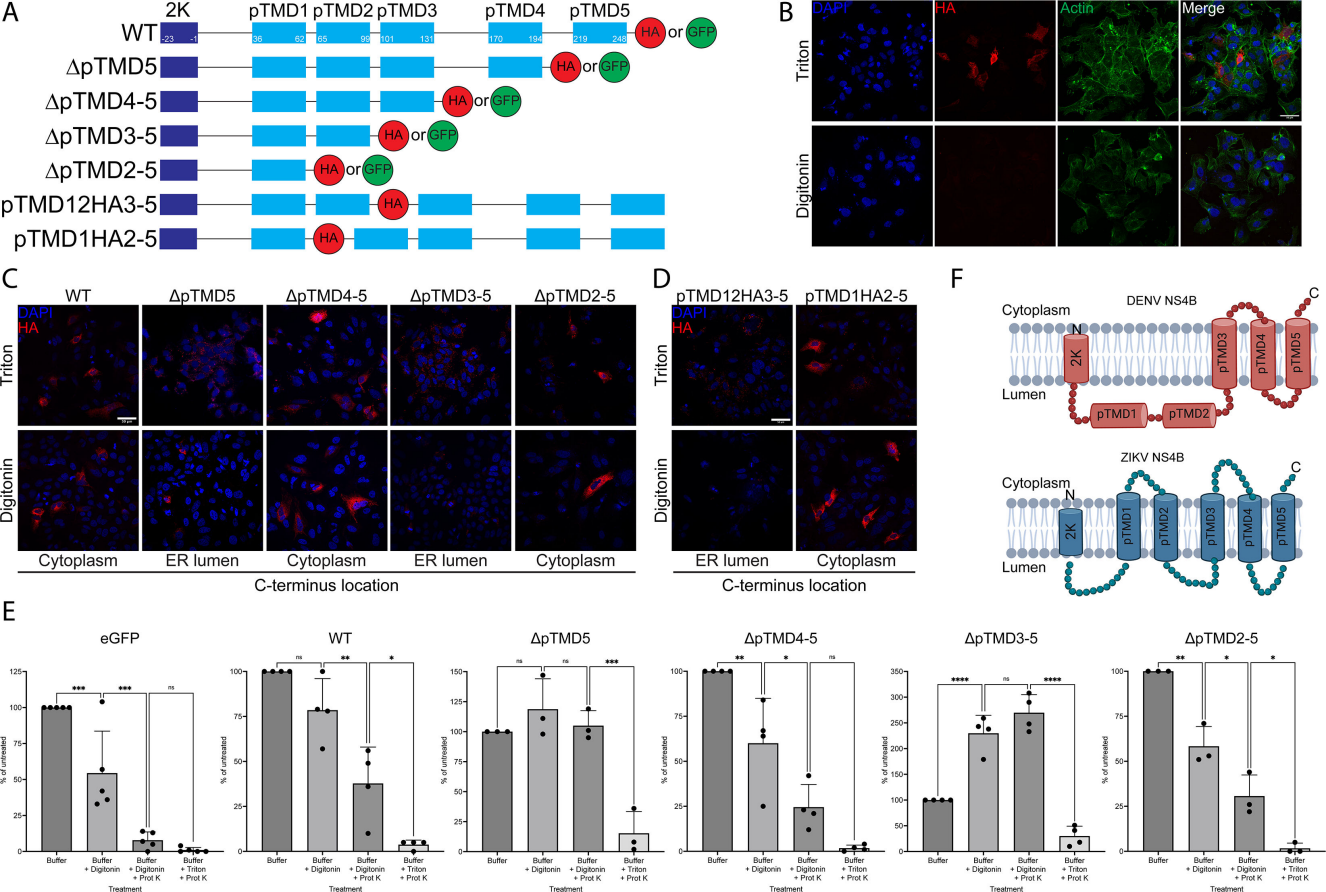
Topology of ZIKV NS4B. (**A**) Schematic of truncation and insertion mutants of ZIKV 2K-NS4B. **B**) Huh7.5.1 cells on coverslips were transfected with a plasmid expressing NHK-HA. Cells were fixed with PFA, permeabilized with Triton X-100 or digitonin, stained with anti-HA and anti-actin antibodies and DAPI, and imaged with a confocal microscope. Micrographs are representative of three independent experiments. Scale bar represents 50 µm. (**C**) Huh7.5.1 cells on coverslips were transfected with plasmids expressing WT or truncated deletion mutations of ZIKV NS4B. Cells were fixed with PFA, permeabilized with Triton X-100 or digitonin, stained with an anti-HA antibody and DAPI, and imaged with a confocal microscope. Micrographs are representative of three independent experiments. Scale bar represents 50 µm. (**D**) Huh7.5.1 cells on coverslips were transfected with plasmids expressing ZIKV NS4B with HA inserted after either pTMD1 or pTMD2. Cells were fixed with PFA, permeabilized with Triton X-100 or digitonin, stained with anti-HA antibody and DAPI, and imaged with a confocal microscope. Micrographs are representative of three independent experiments. Scale bar represents 50 µm. (**E**) 293T cells were transfected with plasmids expressing GFP-tagged WT or truncated deletion mutations of ZIKV NS4B. Live cells were washed with buffer and imaged on a confocal microscope. Cells were then permeabilized with digitonin and imaged. Permeabilized cells were then exposed to proteinase K and imaged. Then, cells were incubated with proteinase K and Triton X-100 and imaged. Total integrated GFP signal from each step was measured and plotted as percentage of buffer-only treatment. Micrographs are representative of three independent experiments. Scale bar represents 50 µm. (**F**) Diagram of experimentally determined DENV (top) (10) and ZIKV (bottom) NS4B topology. Created in BioRender.

To that end, we generated a series of C-terminal HA-tagged truncation mutant constructs of ZIKV 2K-NS4B where each pTMD starting from the C-terminus was sequentially removed starting with ΔpTMD5 and ending with ΔpTMD2-5 (Fig. 3A). We transfected the constructs into Huh7.5.1 cells and fixed the cells with PFA. The coverslips were then differentially permeabilized by treatment with either Triton X-100 (disrupting all membranes) or digitonin (disrupting only the plasma membrane). We then performed immunofluorescence using an anti-HA antibody. By comparing the epitope accessibility across the different permeabilization conditions, we deduced the subcellular location (cytoplasmic or ER luminal) of the C-terminus of each HA-tagged construct. An ER luminal-localized HA tag (NHK-HA) was used a as a control (15). In both treatments, cytoplasmic actin was readily detected, but NHK-HA was only detected in Triton-permeabilized cells (Fig. 3B). We applied this system to the HA-tagged NS4B deletion mutants to determine the C-terminal location of each, and thus could determine whether each predicted TMD crossed the ER membrane. HA signal was similar in the Triton- and digitonin-permeabilized coverslips in the wild-type, ΔpTMD4-5, and ΔpTMD2-5 ZIKV NS4B-transfected cells, suggesting a cytoplasmic location for the HA epitope (Fig. 3C). Conversely, HA signal was starkly reduced in digitonin-treated coverslips in the ΔpTMD5 and ΔpTMD3-5-transfected samples, corresponding with a C-terminus located in the ER lumen for these proteins (Fig. 3C). This implies that all five predicted TMDs cross the ER membrane.

Significant truncations of ZIKV NS4B have the potential to alter its structure. To confirm our ZIKV NS4B topology in the context of the full-length protein, we created constructs with an inserted HA tag immediately after pTMD1 (pTMD1HA2-5) and pTMD2 (pTMD12HA3-5) without any truncation of the remaining pTMDs (Fig. 3A). We then performed the differential permeabilization immunofluorescence experiments on cells transfected with these constructs. Cytoplasmic HA was detected in cells transfected with pTMD1HA2-5 but not pTMD12HA3-5, suggesting that pTMD1 and pTMD2 fully cross the ER membrane, in agreement with the data from the truncation mutants (Fig. 3D).

To further complement our data, we developed an analogous method using GFP-tagged truncation mutations (Fig. 3A) to perform a live cell, fluorescence protease protection assay. In this assay, a field of live 293T cells expressing these proteins (or eGFP as a control) was subjected to a series of detergent and enzymatic treatments while being sequentially imaged with a confocal microscope. First, all cells were treated with digitonin to selectively permeabilize the plasma membrane. Cytoplasmic proteins were then enzymatically digested using proteinase K. Finally, all cellular membranes were permeabilized using Triton X-100, making all proteins accessible to proteinase K digestion. GFP signal was significantly reduced following digitonin permeabilization of the eGFP, ΔpTMD4-5, and ΔpTMD2-5 transfected samples, consistent with some fraction of the protein diffusing out of the cell (Fig. 3E and 1). Conversely, the subcellular localization and lack of decrease of GFP signal intensity after digitonin treatment of WT, ΔpTMD5, and ΔpTMD3-5 indicate that these proteins are bound to a digitonin-resistant membrane (Fig. 3E and 1). There were statistically significant decreases in GFP signal following proteinase K treatment in digitonin permeabilized cells expressing eGFP, WT, ΔpTMD4-5, and ΔpTMD2-5 ZIKV NS4B, suggesting a cytoplasmic localization of the GFP tag (Fig. 3E and 1). In contrast, the GFP signal was sensitive to proteinase K digestion only after treatment with Triton X-100 in the ΔpTMD5 and ΔpTMD3-5 ZIKV NS4B, consistent with GFP localization within the ER lumen (Fig. 3E and 1). These data also suggest that each pTMD does indeed cross the ER membrane.

Interestingly, the data collectively support a topology of ZIKV NS4B differing from that of DENV NS4B (Fig. 3F), where the first two pTMDs do not fully cross the ER membrane. This points to a discrepancy between the computationally predicted topology of ZIKV NS4B (Fig. 1A) and the experimentally determined topology, suggesting that NS4B topology is not conserved across all species of flavivirus (Fig. 3F).

### Characterization of DENV NS4A Y97C NS4B N245Y mutants

**Fig 4:**
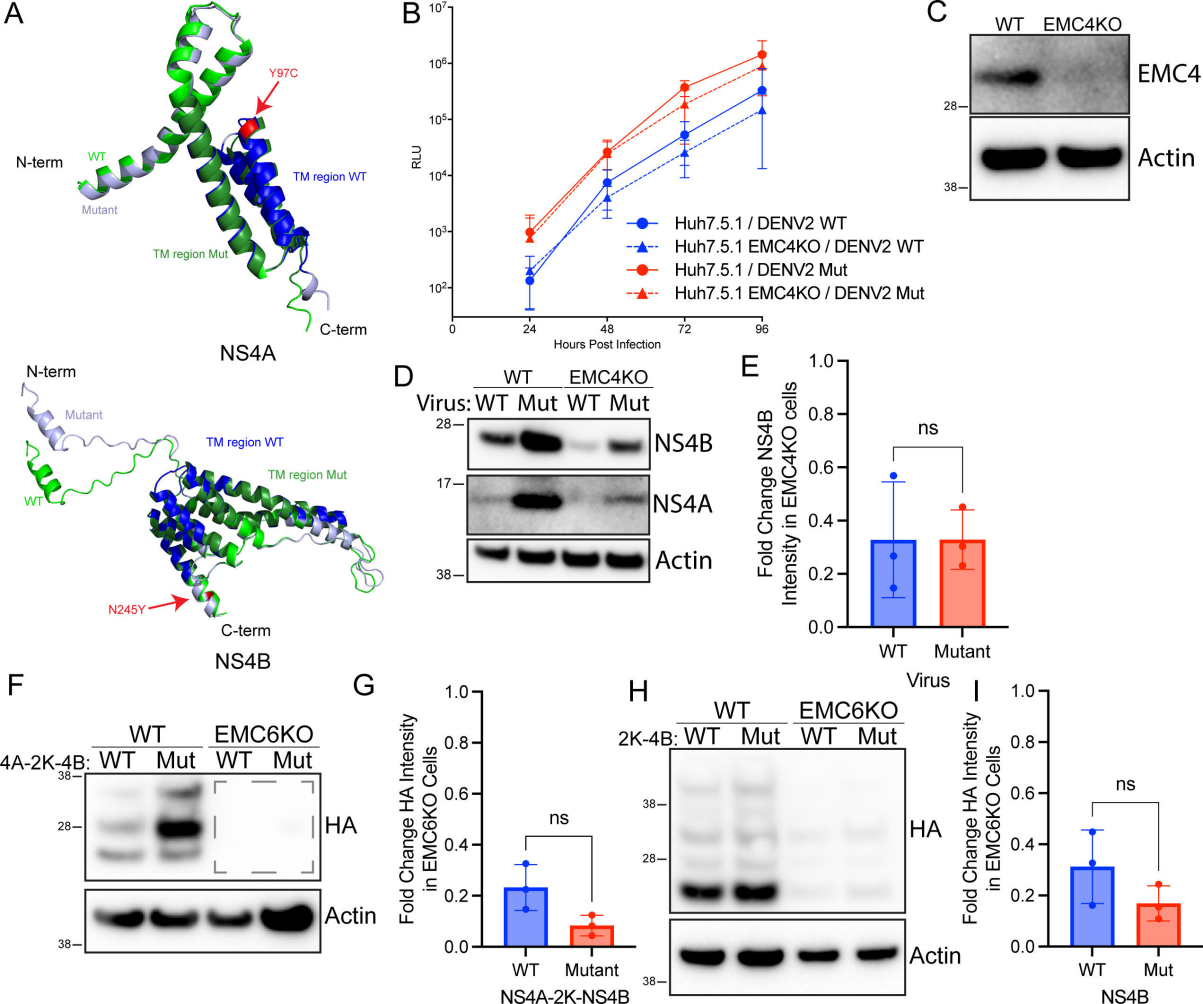
Characterization of potential EMC-independent DENV mutants. (**A**) Alignments of AlphaFold3-predicted structures of WT and putative EMC escape mutants of DENV NS4A and NS4B. Point mutations are indicated in red. (**B**) Wild-type Huh7.5.1 cells or EMC4 KO cells were infected with WT or NS4A Y97C/NS4B N245Y mutant DENV2-Luc reporter viruses at 0.1 MOI. Luciferase activity was measured at the indicated timepoints as a readout of viral infection. Points represent means ± SD of four independent experiments with three technical replicates in each experiment. (**C**) Wild-type Huh7.5.1 cells or EMC4 stable KO cell pools were lysed, and proteins were resolved using SDS-PAGE followed by immunoblotting with the indicated antibodies. (**D**) Wild-type Huh7.5.1 cells or EMC4 stable KO cell pools were infected with WT or NS4A Y97C/NS4B N245Y mutant DENV2-Luc viruses at 0.1 MOI. At 48 hpi, cells were lysed, and proteins were resolved using SDS-PAGE followed by immunoblotting with the indicated antibodies. Blots are representative of three independent experiments. (**E**) Quantitation of D. Bars represent means ± SD. (**F**) Wild-type 293T cells or EMC6 KO cells were transfected to express either WT or mutant (Y97C/N245Y) DENV NS4A-2K–NS4B-HA. Proteins were resolved using SDS-PAGE followed by immunoblotting for the indicated proteins. Blots are representative of three independent experiments. (**G**) Quantitation of F. Dashed lines on F represent quantitation area for KO cells. Bars represent means ± SD. (**H**) Wild-type 293T or EMC6 KO cells were transfected to express either WT or mutant (N245Y) DENV 2K-NS4B-HA. Proteins were resolved using SDS-PAGE followed by immunoblotting for the indicated proteins. Blots are representative of three independent experiments. (**I**) Quantitation of F. Bars represent means ± SD.

A recent report described the identification of a DENV isolate with two amino acid substitutions, one each in NS4A (Y97C) and NS4B (N245Y), that replicated at wild-type levels in EMC-depleted cells (4). Interestingly, the mutation in NS4B, at the far C-terminal end of the protein, was not within the domains (pTMD1 + 2) we had previously shown to be responsible for EMC dependence or, for that matter, in any other pTMDs. As this suggested a model for EMC dependency that was different from those proposed by previous studies of EMC function, we further investigated these mutants to characterize the mechanisms of their escape from EMC dependence. The AlphaFold3-predicted structures of these mutant NS4A and NS4B proteins are quite similar (0.781 Å and 0.174 Å RMSD, respectively) to those of the wild-type proteins, suggesting that there is no dramatic change in structure (Fig. 4A). We assessed the effect of these mutations on viral replication by infecting wild-type and EMC4 knockout Huh7.5.1 cells with wild-type DENV2-Luc wild-type or DENV2-Luc mutant reporter viruses and measuring luciferase activity over 4 days. We generated EMC4 KO cell pools (Fig. 4C) for this experiment to more closely replicate Ngo et al., who used EMC4 KO cells instead of the EMC6 KO cells that we used for our other experiments. While both cell lines infected with the mutant DENV had higher levels of luciferase activity compared with those infected with wild-type virus (Fig. 4B), both the wild-type and mutant viruses displayed statistically indistinguishable fold change decreases (0.53× for wild-type and 0.44× for mutant at 72 hpi) in luciferase activity in EMC4 knockout cells across all timepoints, indicating that the mutations do not confer resistance to EMC knockout.

As the putatively EMC-independent DENV isolate appeared to exhibit EMC dependency in an infection assay, we hypothesized that mutant NS4A and/or NS4B would also display EMC dependency in terms of protein expression. To evaluate this hypothesis, we performed immunoblots for NS4A and NS4B on lysates from wild-type and EMC4 knockout cells infected with DENV2 wild-type or the mutant virus. Expression levels of NS4A and NS4B from both viruses were reduced to a similar degree in EMC knockout cells compared with wild-type controls (Fig. 4D and E), suggesting that the mutations are not sufficient to overcome EMC dependency at the protein expression level.

To further test this in a replication-independent manner, we constructed plasmids expressing HA-tagged wild-type or mutant NS4A (Y97C) and NS4B (N245Y). These constructs were used to transfect wild-type or EMC6 knockout 293T cells, and protein levels were quantified by immunoblot. Both NS4A-2K–NS4B and 2K-NS4B levels were significantly reduced in EMC6 knockout cells compared with wild-type cells (Fig. 4F through I), suggesting that these two amino acid substitutions cannot stabilize NS4A-2K–NS4B or 2K-NS4B in the absence of the EMC. Combined, these results indicate that the Y97C/N245Y substitutions do not confer resistance to EMC dependence for DENV replication or NS4A/NS4B biogenesis.

### Serial passage of ZIKV in EMC knockout cells does not produce EMC-independent isolates

**Fig 5:**
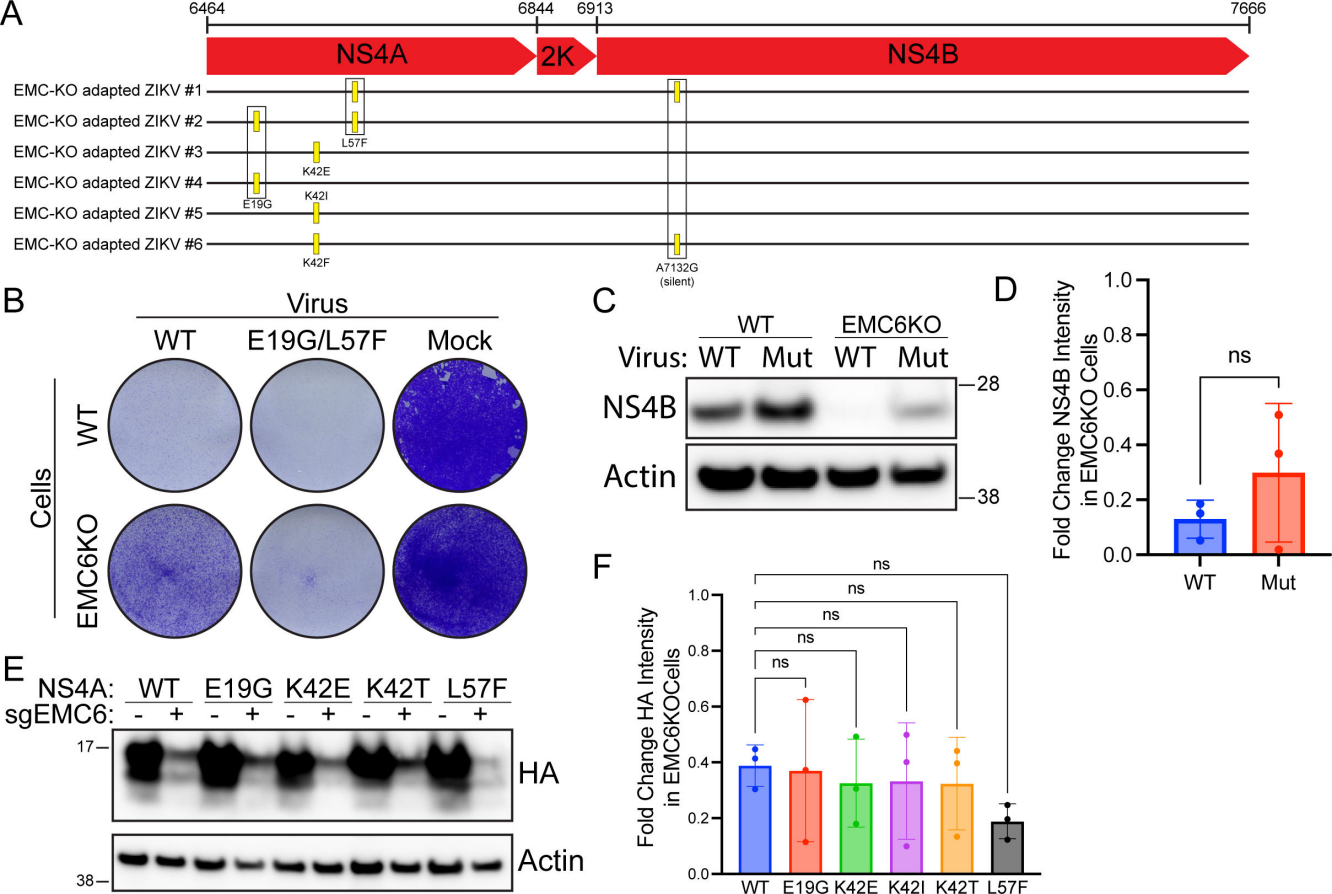
Characterization of potential EMC-independent ZIKV mutants. (**A**) Huh7.5.1 EM6 KO cells were infected with ZIKV PRVABC59 at an initial MOI of 2.2 and serially passaged every 3–4 days. After 12–24 passages, supernatant was collected, RNA was extracted and subjected to RT-PCR to amplify the NS4A-2K–NS4B region. PCR products were sequenced, and SNPs were compared with wild-type genome. (**B**) Wild-type or EMC6 KO Huh7.5.1 cells were infected with WT or NS4A E19G/L57F mutant ZIKV at 0.2 MOI or mock infected. At 72 hpi, cells were fixed with 4% PFA and stained with methylene blue. (**C**) Wild-type or EMC6 KO Huh7.5.1 cells were infected with WT or NS4A E19G/L57F mutant ZIKV at 0.1 MOI. At 48 hpi, cells were lysed, and proteins were resolved using SDS-PAGE followed by immunoblotting with the indicated antibodies. Blots are representative of three independent experiments. (**D**) Quantitation of C. Bars represent means ± SD. (**E**) Wild-type or EMC6 KO 293T cells were transfected with plasmids expressing either WT ZIKV NS4A or the indicated mutant. Proteins were resolved using SDS-PAGE followed by Western blotting for the indicated proteins. Blots are representative of three independent experiments. (**F**) Quantitation of E. Bars represent means ± SD.

At the same time that we began investigating a possible mechanism for the putative EMC-escape DENV mutants, we also performed viral adaptation experiments in ZIKV in parallel. Wild-type ZIKV PRVABC59 was used to infect EMC6 knockout Huh7.5.1 cells in six independent wells, and each was serially passaged every 3 days for 12–24 passages until significant CPE was observed. Since the objective was to identify mutations in NS4A or NS4B that conferred NS4A or NS4B protein stability in EMC knockout cells, the NS4A-2K–NS4B genomic region was sequenced from each independent isolate. All isolates had at least one nonsynonymous mutation in NS4A, and several of these mutations occurred in more than one isolate, suggesting positive selective pressure (Fig. 5A). Three out of six isolates had substitutions at the K42 residue (K42E, K42I, and K42F) located just before the first pTMD. The most common single substitutions in NS4A were E19G (not within a pTMD) and L57A (in pTMD1), which were detected in two isolates each. Interestingly, no NS4B amino acid substitutions were detected in any isolate, although two samples had the same A7132G silent mutation (Fig. 5A). E19G and L57F mutations have been found in patients in Honduras and the United States, respectively, although not together in the same genome. The other mutations have not been reported in NCBI or Nextstrain.

We observed the most CPE with isolate #2 (referred to as ZIKV NS4A E19G/L57F) so it was selected for further analysis. To assess the ability of this isolate to replicate in the absence of EMC, ZIKV NS4A E19G/L57F and the wild-type strain were used to infect wild-type and EMC6 knockout Huh7.5.1 cells for 72 h. To quantify cell death, cells were fixed and stained with methylene blue. As expected, wild-type virus-induced cell death was notably decreased by EMC6 knockout (Fig. 5B). Interestingly, the mutant virus was capable of causing widespread cell death in both wild-type and EMC6 knockout cells (Fig. 5B).

To determine if the E19G/L57F in NS4A conferred EMC independence to NS4A and NS4B, we infected wild-type and EMC knockout Huh7.5.1 cells with wild-type and ZIKV NS4A E19G/L57F and measured viral protein levels by immunoblot (Fig. 5C and D). The decrease in NS4B levels from wild-type to EMC knockout cells was the same across both viruses, indicating that the NS4A E19G/L57F substitutions do not stabilize NS4B in EMC-deficient conditions. To prevent any impact on the mutations on anti-NS4A antibody binding, we constructed HA-tagged constructs expressing each of the NS4A mutations detected in the adaption screen. When transfected into wild-type and EMC6 knockout cells, all mutant constructs demonstrated decreased expression of NS4A, indicating that none of the identified mutations confer NS4A independence from the EMC (Fig. 5E and F).

Combined, our results suggest that while ZIKV isolates adapted to EMC depletion may be able to induce death in EMC-deficient cells, they cannot rescue NS4A and NS4B expression. These results are consistent with our findings on the DENV EMC KO-adapted isolates and are consistent with a very high genetic barrier to EMC knockout.

## DISCUSSION

All flaviviruses are entirely dependent on the ER for all aspects of their lifecycle. Translation of viral proteins, genomic replication of membrane, and assembly of progeny virions all occur at the ER membrane, which is extensively remodeled into structures known as replication organelles to facilitate these processes. While the totality of factors involved in RO formation has yet to be fully described, it has been shown that several viral factors, including proteins NS4A and NS4B, are indispensable for this function.

The EMC has been shown to be a pro-viral factor for several different species of flaviviruses, including DENV, YFV, ZIKV, and WNV (16–20). For ZIKV and DENV, the mechanism of EMC dependence has been proposed to be through support of NS4A and NS4B biogenesis (3, 4). Here, we propose that this mechanism likely extends to WNV and YFV, as we showed that NS4A and NS4B from these flaviviruses also depend on the EMC for effective synthesis. The NS4B proteins from these viruses appear to be ideal clients for the EMC, as each has multipass transmembrane domains with varying degrees of hydrophobicity. Cellular clients of the EMC have been shown to be enriched in these characteristics (21). Interestingly, the determinants of EMC dependency for ZIKV NS4B, which has two pTMDs of reduced hydrophobicity at the C terminal end, appear to be different from those of DENV NS4B, as deletion of pTMDs 1 + 2 or pTMDs 4 + 5 had no effect on ZIKV NS4B protein levels in EMC-depleted cells, in contrast to DENV NS4B where EMC dependency is encoded solely in pTMDs 1 + 2. It is therefore possible that different species of flavivirus NS4B have different determinants of EMC dependency and may therefore interact differently with the EMC during biogenesis.

The four species of NS4B examined in this study had differences in their predicted transmembrane probability plots, suggesting the possibility of variation in their membrane topologies. We experimentally determined that the first two hydrophobic domains of ZIKV NS4B are membrane-spanning, unlike DENV. As both of these topologies largely agree with their TMHMM 2.0 predictions, it suggests that WNV and YFV NS4B might also have differing topologies from DENV NS4B. However, these observations need to be reconciled with the likelihood that all flavivirus NS4B proteins share similar functions in the context of viral replication. One possibility is that the topologies of pTMD1 and pTMD2 may be dynamic depending on the context of expression. For example, it has been previously proposed that whether pTMD5 of DENV NS4B crosses the ER membrane may depend on its association with NS5 at its C-terminus (10, 14).

We also investigated a previous report of an “EMC-independent” isolate of DENV (4), as this appeared to discordant with current models of EMC function, in which the EMC recognizes multipass transmembrane domain proteins and/or marginally hydrophobic domains. The reported NS4B mutation did not occur in the two marginally hydrophobic domains (pTMD1 and pTMD2) that we had previously shown to be responsible for conferring EMC dependence in DENV NS4B (3) nor any of the other TMDs. We were unable to demonstrate the mutant virus’s ability to replicate to wild-type levels in EMC4 KO cells, which we used instead of EMC6 KO to more closely match their experimental conditions. While depletion of EMC6 and other “core” subunits (EMC1, EMC2, EMC3, and EMC5) has been shown to destabilize the entire EMC, previous studies have shown that EMC4 knockdown has limited effect on other EMC subunits (7, 22). As our cells were knockout pools and those of Ngo, et al. were from single-cell clones, it is possible that there are clonal effects that could explain the apparent differences in viral replication. However, we also demonstrated that these substitutions failed to stabilize mutant NS4A and/or NS4B expression in EMC KO cells using immunoblotting, in contrast to Ngo and colleagues who used a dual fluorescence flow cytometry-based assay using constructs that contained NS5 (4). Taken together, our data are more consistent with the two mutations acting as cell culture adaptive mutations rather than these mutations ablating EMC dependency for DENV NS4A and NS4B biogenesis.

We also attempted to identify ZIKV mutations that confer EMC independence. We were able to select for isolates that caused marked CPE in EMC-depleted cells. Sequencing revealed nonsynonymous mutations in NS4A but none in NS4B. None of the mutations were able to stabilize NS4A in the absence of the EMC. Additionally, even in an isolate that caused significant CPE in infected EMC KO cells, NS4B levels were severely diminished. Given that our screen during viral passaging was for the ability to cause CPE, it is most likely that these mutations simply reflect selection for increased CPE or other tissue culture adaptations. Previous work in serially passaging ZIKV in mice has also led to the isolation of the E19G mutation where it has been demonstrated to be associated with increased virulence (23, 24). Collectively, our work with both DENV and ZIKV is consistent with a high genetic barrier to NS4A and NS4B developing independence from the EMC.

In summary, this work underscores the importance of the EMC (biochemically and genetically) to multiple species of flaviviruses. Targeting the EMC-NS4B interaction may have broad anti-flavivirus activity with a high genetic barrier for resistance.

## MATERIALS AND METHODS

### Cell lines

293T, Huh7.5.1, Vero E6 cells and their derivatives were cultured in DMEM containing 10% FBS and 100 U/mL penicillin–streptomycin in a 37°C incubator with 5% CO_2_.

### Viruses

DENV2-Luc wild-type and mutant plasmids were a gift from Andreas Puschnik. Viral RNA was generated by *in vitro* transcription with HiScribe T7 mRNA Kit with CleanCap Reagent AG (NEB) and cleaned up with Quick-RNA Miniprep (Zymo) per manufacturer’s instructions. RNA was transfected into Vero E6 cells using TransIT-mRNA transfection reagent (Mirus Bio). Supernatant was harvested 5–7 days post-transfection, filtered, and stored at −80°C in single-use aliquots. ZIKV PRVABC59 stock was a gift from Kathy Spindler (University of Michigan). Viral stocks were generated by infecting Vero E6 cells for 3–5 days and harvesting filtered supernatant.

### Titration of DENV and ZIKV

Viral stocks were serially diluted and used to infect VeroE6 cells in a 96-well plate for 3 days. Cells were fixed with 1:1 acetone:methanol for 20 min at −20°C. Cells were then incubated in blocking buffer (5% FBS, 0.05% Triton X-100 in PBS) for 30 min, incubated with 4G2 antibody diluted in blocking buffer for 50 min, washed with PBS, incubated with Alexa 488 anti-mouse secondary antibody for 40 min, washed with PBS, stained with DAPI, and washed with PBS again before infected cells were counted on an epifluorescence microscope and FFU/mL was determined.

### Infections

Virus stocks were diluted into DMEM containing 2% FBS and 100 U/mL penicillin–streptomycin at the indicated MOI, applied to Huh7.5.1 cells, and incubated in a 37°C incubator with 5% CO_2_ until the indicated timepoint.

### Transfections

DNA transfections were performed with FuGENE HD Transfection Reagent (Promega) per manufacturer’s instructions.

### Immunoblotting

At 24 h post-transfection, cells were lysed with NP-40 Lysis Buffer (20 mM Tris pH 8, 137 mM NaCl, 2 mM EDTA, 1% NP-40, 10% glycerol) supplemented with 1× Halt Protease Inhibitor (Thermo Fisher). Lysates were clarified by centrifugation at 10,000×*g* for 10 min at 4°C. Clarified lysates were either processed immediately or stored at −80°C until use. LDS sample buffer (Thermo Fisher) and beta-mercaptoethanol (Millipore Sigma) were added to lysates before incubating at 70°C or 37°C (for NS4A blots) for 10 min. Samples were resolved on 4%–12% NuPAGE Bis-Tris mini gels (Invitrogen) in 1× MES Buffer (Invitrogen). Proteins were transferred to a 0.45 µm PVDF membrane (Millipore). After transfer, membranes were blocked for 30 min in 5% nonfat dry milk in Tris-buffered saline with Tween 20 (TBST). Membranes were sequentially incubated overnight at 4°C with primary antibodies diluted in blocking buffer, washed, and then incubated for 1 h at RT secondary antibodies. Proteins were detected by the addition of SuperSignal West Femto (Thermo Fisher) substrate and imaged on a G:BOX imager (Syngene). Quantitation of chemiluminescent signals was performed using GeneTools software (Syngene).

### Antibodies

Antibodies used in this study are listed in Table 1.

**TABLE 1:** Antibodies

Antigen	Supplier	Catalog #	Dilution
HA	Cell Signaling Technologies	C29F4	1:2,000 (WB), 1:300 (IF)
FLAG	Sigma	F1806	1:1,000
DENV NS4A	Genetex	GTX132069	1:1,000
DENV NS4B	Genetex	GTX103349	1:2,000
ZIKV NS4A	Genetex	GTX133704	1:1,000
ZIKV NS4B	Genetex	GTX133321	1:1,000
β-Actin	Sigma	A1978	1:10,000, 1:300 (IF)
GFP	Cell Signaling Technologies	2956	1:1,000
Mouse-HRP conjugated	Invitrogen	32430	1:500
Rabbit-HRP conjugated	Invitrogen	32460	1:500
Mouse Alexa Fluor 488	Invitrogen	A-11001	1:500
Rabbit Alexa Fluor 568	Invitrogen	A-11011	1:500
Flavivirus E	ATCC	D1-4G2-4-15	1:10

### Immunofluorescence

Huh7.5.1 cells were plated on poly-D-lysine coated coverslips and transfected with HA-tagged ZIKV NS4B constructs. At 24 h after transfection, cells were fixed with 4% PFA for 15 min at room temperature and then moved to a humid chamber. Cells were then permeabilized with 0.2% Triton X-100 for 5 min or 5 μg/mL digitonin for 30 min. After permeabilization, coverslips were incubated in the corresponding blocking buffer (10% FBS in PBS supplemented with 0.05% Triton X-100, 5 μg/mL digitonin, or no detergent) for 30 min. Coverslips were incubated in primary antibody diluted in blocking buffer for 60 min before being washed three times in PBS for 5 min and then incubated in secondary antibody diluted in blocking buffer for 60 min at room temperature. After washing with PBS, coverslips were incubated for 5 min at room temperature in 300 nM DAPI (Thermo Fisher) before two more washes with PBS and were mounted on glass slides with ProLong Gold (Invitrogen). Coverslips were imaged with a Nikon A1R or Zeiss LSM 800 confocal microscope with image processing using FIJI v2.15.1 (25).

### Serial passage of ZIKV passaging

Six independent wells of EMC6 KO Huh7.5.1 cells were infected with ZIKV PRVABC59 at an MOI of 2.2 for 3 days. Each supernatant was independently filtered and passaged 1:4 onto new EMC6 KO Huh7.5.1 cells for 3 days. This was repeated 12–24 times until significant CPE could be detected at 2 dpi with a 1:2,000 passage. Then, the viral RNA was extracted, NS4A-2K–NS4B was amplified by PCR, and sequenced.

### Fluorescence protease protection assay

293T cells were plated in dishes containing a poly-D-lysine-coated coverslip and transfected with GFP-tagged ZIKV NS4B constructs. At 24 h after transfection, cells were washed three times with KMH buffer (110 mM potassium acetate, 3 mM magnesium acetate, 20 mM HEPES-NaOH, pH 7.2) and imaged on Zeiss LSM 800 confocal microscope. The cells were then incubated in KMH buffer supplemented with 30 µM digitonin for 5 min, washed with the same buffer, and imaged again. Next, the cells were incubated in KMH buffer supplemented with 30 µM digitonin and 50 µg/mL proteinase K for 5 min and imaged. Finally, the cells were incubated in KMH buffer supplemented with 0.3% Triton X-100 and 50 µg/mL proteinase K for 2 min and imaged again. Total integrated density of GFP signal was measured in FIJI for each step and was calculated as a percentage of untreated cells.

### Protein structure predictions

Amino acid sequences of various flavivirus NS4A/NS4B were input into AlphaFold v2.3.1 (for flavivirus species comparison) or AlphaFold 3 (for DENV mutant NS4A/NS4B comparison). The resulting PDB files were analyzed and aligned with PyMOL v3.0.3.

## Data Availability

No genomic, proteomic, or other large data sets were generated in this work for deposition in public repositories. No new software or computer algorithms were generated.

## SUPPLEMENTAL MATERIAL

Figure S1 jvi.01443-24-s0001.tif Fluorescence protease protection assay.

Supplemental legend jvi.01443-24-s0002.docx Legend for Fig. S1.

ASM does not own the copyrights to Supplemental Material that may be linked to, or accessed through, an article. The authors have granted ASM a non-exclusive, world-wide license to publish the Supplemental Material files. Please contact the corresponding author directly for reuse.
