# Supplementary figures and images for: Dengue and Zika virus NS4B proteins differ in topology and in determinants of ER membrane protein complex dependency
Source: J Virol. 2024 Dec 31;99(2):e01443-24. doi: 10.1128/jvi.01443-24 (PMC11852961; doi:10.1128/jvi.01443-24)

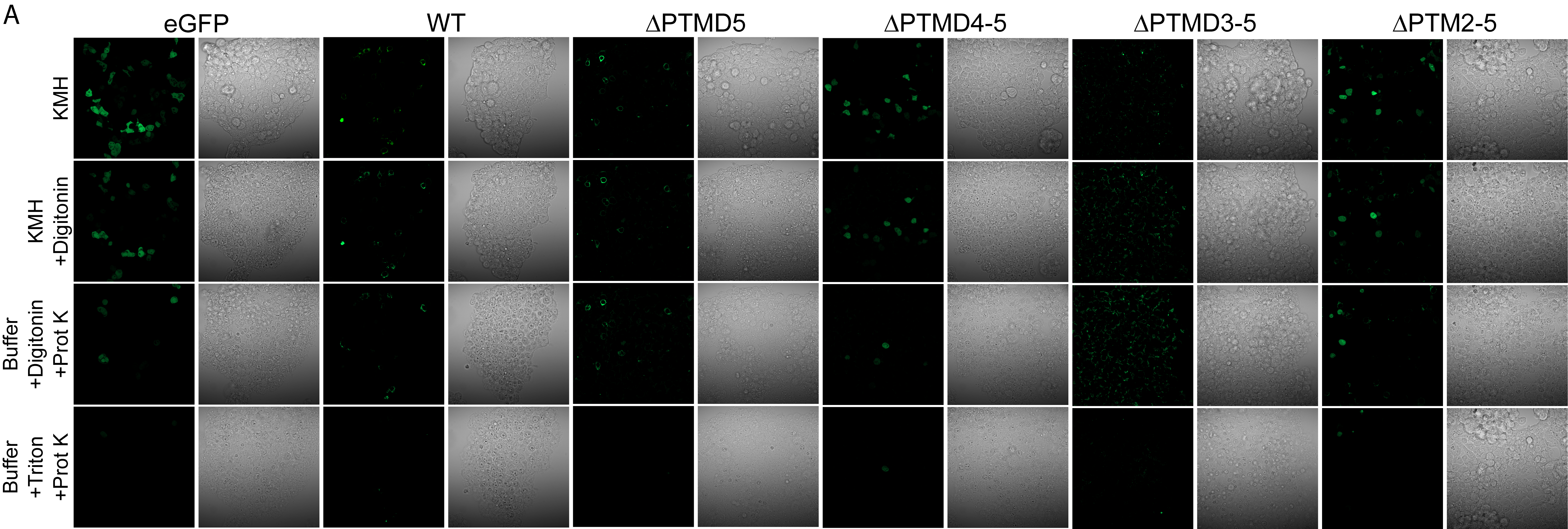

Supplement: Figure S1 — Fluorescence protease protection assay. [file jvi.01443-24-s0001.tif]
