# Supplementary material for: Dengue and Zika virus NS4B proteins differ in topology and in determinants of ER membrane protein complex dependency
Source: J Virol. 2024 Dec 31;99(2):e01443-24. doi: 10.1128/jvi.01443-24 (PMC11852961; doi:10.1128/jvi.01443-24)
Supplement: Supplemental legend — Legend for Fig. S1. [file jvi.01443-24-s0002.docx]

**Figure S1: Fluorescence Protease Protection assay**. 293T cells were transfected with plasmids expressing GFP-tagged WT or truncated deletion mutations of ZIKV NS4B. Cells were washed with KMH buffer and a field was imaged on a confocal microscope. Cells were then permeabilized with digitonin and imaged. Permeabilized cells were then exposed to proteinase K and imaged. Then cells were incubated with proteinase K and Triton X-100 and imaged. Micrographs are representative of at least three independent experiments. Scale bar represents 50 µm.
